# Birthday Honours

**Published:** 1917-06-09

**Authors:** 


### BIRTHDAY HONOURS.

A large number of honours have been conferred in the Birthday list on medical men, but of twenty-five new baronets only one, in the person of Sir Frederick Taylor, President of the Royal College of Physicians of London, is a member of the medical profession. Sir Frederick is consulting physician to Guy's Hospital, and the author of the very well-known text-book of medicine, which has passed through many editions. There are three new medical knights: Sir Herbert Furnival Waterhouse, the senior surgeon to Charing Cross Hospital and a member of the Council of the Royal College of Surgeons. He has for many months been surgeon to the British Hospital in Petrograd, but has now returned to London. Sir Robert Jones is surgeon to the Royal Southern Hospital, Liverpool, and to the Royal Liverpool Country Hospital for Children. He is famous for his work in orthopædic surgery. Sir T. Kennedy Dalziel is a well-known Glasgow surgeon, and is a lecturer on clinical surgery at the University there. He is consulting surgeon to the Royal Hospital for Sick Children, Glasgow. The following is a full list of the remaining Honours:—

### Order of the Bath.

*Members of the Military Division of the Second Class, or Knights Commanders*:—Col. J. Magill, M.D., and Lieut.-Col. J. J. Freyer, M.D., I.M.S. (ret.).

*Members of the Military Division of the Third Class, or Companions*:—Lieut.-Col. and Brevet-Col. Sir B. G. Seton, Bart., I.M.S.; Col. D. Wardrop,, A.M.S.; Temp. Col. J. Galloway, A.M.S.; Surg.-Lieut.-Col. (Hon. Surg.-Col.) R. J. Reece, H.A.C.; Surg.-Lieut.-Col. Sir W. R. Crookes-Lawless (empld. R.A.M.C.).

*Members of the Civil Division of the Third Class, or Companions*:—Temp. Surg.-Gen. Sir William Macewen, R.N., and Temp. Surg.-Gen. G. R. Turner, R.N.

*Member of the Civil Division of the Second Class, or Knight Commander*:—E. A. Gowers, Chief Inspector, National Health Insurance Commission.

### Order of St. Michael and St. George.

*Members of the Second Class, or Knights Commanders*:—Surg.-Gen. Sir A. T. Sloggett; Major-Gen. A. L. Lynden-Bell; Surg.-Gen. T. P. Woodhouse; Col. R. M. Campbell, I.M.S.

*Members of the Third Class, or Companions*:—Col. D. M. O'Callaghan, A.M.S.; Col. A. W. Bewley, A.M.S.; Lieut-Col. G. Scott, late R.A.M.C.; Lieut.-Col. A. Hosie, late R.A.M.C.; Lieut.-Col. (temp. Col.) J. C. Connor, R.A.M.C.; Temp. Lieut. Col. A. D. Milne, E.Afr.M.S.; Major (temp. Lieut.-Col.) E. J. C. Thompson, R.A.M.C.; Col. R. E. Roth, A.M.C.; Lieut.-Col. J. N. MacLeod, I.M.S.; Lieut.-Col. D. Harvey, R.A.M.C.; Major P. S. O'Reilly, R.A.M.C.; Col. R. J. Millard, A.M.C.; Lieut.-Col. W. T. Hatward, A.M.C.; Lieut-Col. T. E. V. Hurley, A.M.C.; Col. C. A. Hodgetts, Can.A.M.C.

The following promotions have also been announced:—Brevet-Colonels: Lieut.-Col. S. L. Cummins, R.A.M.C.; Lieut.-Col. (temp. Col.) G. T. K. Maurice, R.A.M.C.

### Companions of the Distinguished Service Order.

Major (temp. Lieut.-Col.) D. Ahern, R.A.M.C.; Temp. Capt. P. H. Bahr, R.A.M.C.; Lieut.-Col. H. J. K. Bamfield, I.M.S.; Lieut.-Col. (temp. Col.) A. W. N. Bowen, R.A.M.C.; Capt. F. Casement, R.A.M.C.; Major (temp. Lieut.-Col.) B. H. V. Dunbar, R.A.M.C.; Temp. Capt. J. J. Dwyer, R.A.M.C.; Capt. (acting Lieut.-Col.) A. D. Fraser, R.A.M.C.; Major G. E. Gask, R.A.M.C.; Lieut.-Col. (acting Col.) J. G. Gill, R.A.M.C.; Lieut.-Col. G. H. Goddard, R.A.M.C.; Capt. J. Golding, R.A.M.C.; Major (temp. Lieut.-Col.) G. H. L. Hammerton, R.A.M.C.; Major E. T. Harris, I.M.S.; Lieut.-Col. (acting Col.) H. Herrick, R.A.M.C.; Lieut.-Col. C. H. Howkins, R.A.M.C.; Lieut. N. S. Jatar, I.M.S.; Capt. H. Lightstone, R.A.M.C.; Capt. (acting Lieut.-Col.) A. S. Littlejohns, R.A.M.C.; Temp. Lieut.-Col. D. D. Logan, R.A.M.C.; Lieut.-Col. (temp. Col.) P. MacKessack, R.A.M.C.; Capt. (acting Lieut.-Col.) G. Mackie, R.A.M.C.; Capt. (acting Lieut.-Col.) R. Magill, R.A.M.C.; Temp. Capt. J. R. Marrack, R.A.M.C.; Lieut.-Col. E. W. P. V. Marriott, R.A.M.C.; Capt. (acting Lieut.-Col.) C. R. M. Morris, R.A.M.C.; Capt. (acting Lieut.-Col.) K. D. Murchison, R.A.M.C.; Lieut.-Col. (temp. Col.) S. de C. O'Grady, R.A.M.C.; Major J. S. Pascoe, R.A.M.C.; Major (temp. Lieut.-Col.) J. E. Powell, R.A.M.C.; Lieut.-Col. (temp. Col.) M. MacG. Rattray, R.A.M.C.; Major (temp. Lieut.-Col.) H. Rogers, R.A.M.C.; Major (temp. Lieut.-Col.) D. Rorie, R.A.M.C.; Temp. Capt. (Acting Lieut.-Col.) L. D. Shaw, R.A.M.C.; Major G. F. Sheehan, R.A.M.C.; Major S. B. Smith, R.A.M.C.; Capt. (temp. Major) R. S. Taylor, R.A.M.C.; Temp. Capt. R. B. Wallace, R.A.M.C.; Capt. (acting Lieut.-Col.) A. G. Wells, R.A.M.C.; Major (temp. Lieut.-Col.) R. M. West, R.A.M.C.; Major W. J. Weston, R.A.M.C.; Lieut.-Col. E. A. Wraith, R.A.M.C.; Major (temp. Lieut.-Col.) T. J. Wright, R.A.M.C.; Major G. C. Byrne, A.A.M.C.; Major G. E. Cole, A.A.M.C.; Lieut.-Col. (temp. Col.) R. B. Huxtable, A.A.M.C.; Capt. A. H. Powell, A.A.M.C.; Major R. W. W. Walsh, A.A.M.C.; Major R. M. Gorssline, Can.A.M.C.; Lieut.-Col. W. B. Hendry, Can.A.M.C.; Lieut.-Col. C. C. F. McGuffen, Can.A.M.C.; Lieut.-Col. R. L. Girdwood, S.A.M.C.; Lieut.-Col. H. W. Vaughan-Williams, S.A.M.C.

*Colonial Office List.—Order of St. Michael and St. George*:—C. M. G. Frederick Truby King, M.B., Medical Superintendent, Seacliff Mental Hospital, New Zealand.

### Knights Bachelor.

The honour of Knight Bachelor was conferred on the following gentlemen:—The Hon. Edward Miller, of Melbourne, Treasurer for the Australian Branch of the British Red Cross Society; Edward Charles Stirling, M.D., Sc.D., C.M.G., Professor of Physiology in the University of Adelaide, for his services to science in Australia.

